# Coronary Atherosclerosis Assessment by Coronary CT Angiography in Asymptomatic Diabetic Population: A Critical Systematic Review of the Literature and Future Perspectives

**DOI:** 10.1155/2018/8927281

**Published:** 2018-01-15

**Authors:** Andrea Igoren Guaricci, Delia De Santis, Mariangela Carbone, Giuseppe Muscogiuri, Marco Guglielmo, Andrea Baggiano, Gaetano Serviddio, Gianluca Pontone

**Affiliations:** ^1^Institute of Cardiovascular Disease, Department of Emergency and Organ Transplantation, University Hospital Policlinico of Bari, Bari, Italy; ^2^Department of Medical and Surgical Sciences, University of Foggia, Foggia, Italy; ^3^Section of Diagnostic Imaging, Interdisciplinary Department of Medicine, University Hospital Policlinico of Bari, Bari, Italy; ^4^Centro Cardiologico Monzino, IRCCS, Milano, Italy; ^5^CMO Torre Annunziata, Napoli, Italy

## Abstract

The prognostic impact of diabetes mellitus (DM) on cardiovascular outcomes is well known. As a consequence of previous studies showing the high incidence of coronary artery disease (CAD) in diabetic patients and the relatively poor outcome compared to nondiabetic populations, DM is considered as CAD equivalent which means that diabetic patients are labeled as asymptomatic individuals at high cardiovascular risk. Lessons learned from the analysis of prognostic studies over the past decade have challenged this dogma and now support the idea that diabetic population is not uniformly distributed in the highest risk box. Detecting CAD in asymptomatic high risk individuals is controversial and, what is more, in patients with diabetes is challenging, and that is why the reliability of traditional cardiac stress tests for detecting myocardial ischemia is limited. Cardiac computed tomography angiography (CCTA) represents an emerging noninvasive technique able to explore the atherosclerotic involvement of the coronary arteries and, thus, to distinguish different risk categories tailoring this evaluation on each patient. The aim of the review is to provide a wide overview on the clinical meaning of CCTA in this field and to integrate the anatomical information with a reliable therapeutic approach.

## 1. Introduction

Diabetes mellitus (DM) is a major public health problem, the incidence of which seems to be drastically increased and will grow in the next years [1, 2]. Many studies in the literature showed a clear correlation between DM and risk of coronary heart disease (CAD). [3–5]. Moreover, compared with matched nondiabetic individuals, patients with diabetes has a higher prevalence, extent, and severity of CAD [6]. On the basis of these considerations and on the beneficial removal of the risk factors on progression of atherosclerotic disease, early detection of diabetic patients at increased risk of adverse cardiac events is crucial.

Coronary computed tomography angiography (CCTA) is an emerging noninvasive technique for the evaluation of coronary stenosis and for the characterization of the atherosclerotic plaques [7–9]. However, although the diagnostic accuracy and prognostic value of CCTA have been largely proved in symptomatic low-intermediate patients [10–13], its role in the asymptomatic and diabetic individuals is still widely debated [14, 15]. The American Diabetes Association and American Heart Association recently issued a joint statement that urges the identification of asymptomatic patients with subclinical CAD in whom more aggressive lifestyle or treatment changes would allow prevention of progression of the disease and reduce future clinical events [16] In the light of this, the objective of our review is to assess the rationale and effectiveness of CAD screening in asymptomatic diabetic patients by CCTA, providing future perspectives on potentiality of this emerging noninvasive imaging technique.

## 2. Diabetes Mellitus as Coronary Risk Equivalent: An Unsolved Matter

CAD represents the main cause of mortality and morbidity in patients affected by DM, which was considered as a “coronary risk” equivalent [17, 18]. The validated correlation between DM and increased risk of CAD sparked a vivacious debate in the scientific community about the appropriateness of considering the diabetics as patients affected by CAD by default. The consideration of the DM as a “coronary equivalent,” however, has a remarkable role because it implies a very aggressive treatment with significant healthcare costs, possible lack of patient's compliance, and risk of adverse effects.

In order to weigh up the risk of CAD in the diabetic population compared to nondiabetic population, Haffner et al. [19] compared the incidence of AMI in 1373 diabetic patients and 1059 nondiabetic subjects in the Finnish population and followed them up for 7 years. The study showed that previous AMI had a substantial role in determination of second AMI, stroke, and cardiovascular death. AMI incidence in nondiabetic population was 18.8% in the population with prior AMI and, conversely, 3.5% in nondiabetic population without prior AMI. In parallel, in diabetes group, the incidence of AMI was 45.0% in the population with prior AMI and 20.2% in the population without prior AMI. On the other hand, nondiabetic patients without previous AMI showed better survival. The substantial novelty of the study is finding a similar incidence of cardiovascular events in the group of 890 patients with DM without prior AMI and in the group of 69 patients without DM but with prior AMI, in a follow-up of 7 years. This correlation is unchanged even after adjustment for demographic variables (age and gender) and other cardiovascular risk factors (smoking, hypertension, lipid profile). On the basis of these results, the authors affirmed the negative impact of DM on coronary perfusion because diabetic patients with no known history of CAD presented the same risk of cardiovascular death as patients without DM but with prior AMI. For this reason, Haffner considered DM as an equivalent of CAD, implying an increase of 20% in the 10-year cardiovascular risk of adverse events. This result suggests and encourages the treatment of all diabetic patients, as if they were really affected by known CAD [20]. Although recent studies confirmed and supported the consideration of “coronary risk equivalency” [21, 22], other bodies of evidence seem to reconsider this assumption, suggesting the identification of different classes of risk [23, 24]. A meta-analysis published in 2009 [25] compared the total risk of coronary events in diabetic patients without previous AMI and nondiabetic patients with previous AMI. This meta-analysis evaluated 13 studies including 45.108 patients with a mean follow-up of 13.4 years and a mean age of the enrolled subjects between 25 and 84 years. 2603 CHD events were found in diabetic population with no previous AMI; on the other hand 3927 events were recorded in the nondiabetic population with prior AMI. This work showed that diabetic patients without previous AMI presented a 43% lower risk of developing coronary events compared with nondiabetic patients with prior AMI (summary odds ratio 0.56, 95% confidence interval 0.53–0.60). This result suggests that, although DM is an important risk factor for the development of cardiovascular adverse events, it cannot be considered as a “coronary risk equivalent.” Recently, Rana et al. [26] have studied a large cohort of 1.586.081 adults, admitted to the Kaiser Permanente Northern California healthcare system, aged between 30 and 90 years with a 10-year follow-up. The study compared the risk of adverse cardiac events in the population divided into 4 groups according to the presence of DM and coronary heart disease (CHD). The study confirmed that the sole presence of prior CHD is associated with an almost twofold increased risk of CHD, compared to the presence of sole DM (12.2 versus 22.5 per 1000 person-years), suggesting that the DM is an additional risk factor rather than a trigger in the progression of CAD. Remarkably, only when diabetes was present for more than 10 years, the risk of future CHD for patients with diabetes was similar to that for those with previous CHD. Of note is that, although the Adult Treatment Panel (ATP) III guidelines in 2001 recommended lifestyle and therapeutic primary prevention in diabetics [20], subsequent ACC/AHA American guidelines on the individual risk assessment reduced the DM role in the progression of atherosclerotic disease, on the basis of these new scientific bodies of evidence in the literature [27]. Therefore, there is no scientific evidence to support an aggressive therapeutic strategy with statins and aspirin in all patients with DM, but only in diabetic patients at high risk, in order to reduce cardiovascular mortality. On the basis of this important meta-analysis, it is crucial and essential to identify diabetic patients at high risk of adverse coronary events, worthy of an adequate aggressive therapy with statins and aspirin.

## 3. Standard Diagnostic Approach to Asymptomatic Diabetic Patient

In view of the high prevalence of CAD and the nonnegligible autopsy rates of silent coronary ischemia in diabetic patients due to prevalent neuropathy [28], noninvasive stress imaging could be useful in the prognostic stratification of asymptomatic diabetic patients in order to minimize vascular consequences of chronic hyperglycemia and optimize therapeutic approach.

**Figure 1:**
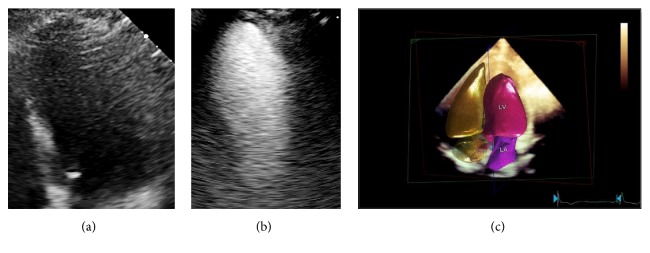
Dobutamine stress echocardiography in patient affected by diabetes mellitus (DM) with chronic pulmonary obstructive disease (COPD) and poor acoustic window. (a) Apical four-chamber view of left ventricle. (b) The use of ultrasound contrast agent definitively allows an acceptable imaging quality of the endocardial border. (c) 3D heart model is imaged. It represents a chamber quantification using a model-based segmentation algorithm for achieving a more accurate functional assessment.

Detecting CAD in patients with diabetes is challenging [29]. The involvement of small vessels due to metabolic abnormalities and the diffuse nature of the disease limit the reliability of cardiac stress tests for detecting myocardial ischemia [17], further worsened by the comorbidities (Figure 1). In addition, the silent fashion of CAD due to the high threshold for pain reduces the sensitivity of clinical risk assessment [30].

Exercise electrocardiography (EKG) is the most used noninvasive technique for the diagnostic and prognostic evaluation of nondiabetic patients with known or suspected CAD. Unfortunately, the accuracy of exercise EKG is reduced in the diabetic population. The poor response in terms of pressure and heart rate increasing during exercise, the high incidence of silent myocardial ischemia and microvascular disease, the alterations of impulse conduction due to visceral neuropathy, the presence of baseline ST-segment abnormalities, left ventricular hypertrophy, and the impaired exercise capacity due to peripheral vascular disease limited the diagnostic and prognostic value of exercise EKG. Given the limited accuracy of exercise EKG stress in diabetic patients, myocardial stress imaging tests have been proposed [31] also thanks to their ability to identify changes in regional contractility depending on the location and size of the ischemic area. In asymptomatic diabetic patients, the sensitivity and specificity of stress echocardiography in the diagnosis of CAD are reported to be 81% and 85%, respectively [32].

Stress echocardiography allows identifying, in the absence of signs of stress ischemia, patients at low risk of developing adverse cardiac events [33, 34]. Two studies conducted on diabetic sample revealed a small number of false negatives in the identification of CAD, sustaining good efficacy of stress echocardiography in identifying low risk diabetic patients to develop cardiac events [35, 36]. Few years later, Kamalesh et al. [37] studied the incidence of adverse cardiac events in a diabetic and nondiabetic population with absence of signs of inducible ischemia as assessed by stress echocardiography and followed them up for 25 ± 7 months. The study showed that diabetic patients, compared to nondiabetic ones, had a higher incidence of cardiac events (19% versus 9.7%, *p* = 0.03), worse event-free survival (*p* = 0.03), and a greater number of nonfatal MI events (6.7% versus 1.4%, *p* < 0.05). The study revealed also that the history of CAD was the only predictor of adverse cardiac events (*R* = 0.18, *p* < 0.05). On the basis of these results, Kamalesh concluded that diabetic patients with negative stress echocardiogram have more risk for adverse cardiac events compared to nondiabetic patients. The cause of this substantial difference could be explained by the greater tendency of diabetic patients to have distal CAD, slightly detectable by stress echocardiography. Moreover, diabetic patient presents an alteration of the coagulation pattern, intense platelet activity, and reduced fibrinolysis which, together with the recognized autonomic dysfunction, most frequently predispose the patient to coronary occlusion [38]. On the same line, Cortigiani et al. [39] showed that a negative, nonischemic stress test in the diabetic population, particularly in the subset of patients aged >65 years, is associated with an increased risk of developing adverse cardiac events when compared to nondiabetic subjects of the same age. Confirming these results, other studies showed an annual incidence of adverse cardiac events in diabetic patients with normal stress test equal to 3–6%, about twice that in nondiabetic patients with normal stress test [40, 41]. On the other hand, some large studies assessed the prognostic value of single-photon emission computed tomography imaging in patients with DM and, importantly, also in this scenario the event rate was higher compared with the control population, even in presence of a normal scan [29, 42].

On the basis of these considerations, although exercise stress testing and myocardial perfusion imaging remain important techniques for risk assessment and prognosis of CAD in asymptomatic diabetic patients, presence of confounding factors, such as autonomic dysfunction, multivessel disease, EKG abnormalities and interpretative difficulties, peripheral artery disease, and the need for polypharmacotherapy, could compromise the diagnostic efficacy explaining why their role remains controversial [43].

## 4. Calcium Scoring

Coronary artery calcium score (CACS) is widely considered a marker of subclinical atherosclerosis, validated in asymptomatic patients [47]. Extent of CACS, in fact, well correlates with the vascular atherosclerotic involvement and the probability of adverse cardiac events in the general population [48–50]. Although the latest European guidelines on cardiovascular prevention [51] suggested evaluation of the CACS only in diabetic patients with high or very high cardiovascular risk (score > 5% and score > 10%), the latest American guidelines for risk stratification in patients with CAD recommended an “appropriate” use of CACS and CCTA in asymptomatic patients with high global risk [52].

Type 2 DM patients have higher values of CACS when compared with the general population [53]. The mechanisms responsible for the extensive intracoronary calcium accumulation in diabetic patients are multifactorial and not completely understood. Previous studies revealed that the increased production of advanced glycation end-products induces the overexpression of genes and enzymes involved in active calcification of the coronary plaque [54]. Coronary artery calcium scoring (CACS) has been proposed as a first-line test for CAD in patients with diabetes [55] since it was widely demonstrated that it has higher capability with respect to conventional cardiovascular risk factors for predicting silent myocardial ischemia and short-term outcome [56]. Numerous studies showed that higher values of CACS in diabetic patients with metabolic syndrome are closely associated with increased prevalence of ischemia, adverse cardiac events, AMI, and mortality [57–60].

Notwithstanding, a significant percentage of patients with DM have very low or zero CACS, with a better long-term prognosis, revealing that DM is not an equivalent of coronary risk. Raggi et al. documented a high proportion of asymptomatic patients with DM (39%) with CACS < 10 [61]. In this study the authors confirmed a significant correlation between CACS and DM (*p* = 0.00001), indicating that each increase of CACS correlates with an increase in mortality in diabetic and nondiabetic patients. However, diabetic patients without known CAD showed similar survival to patients without DM and intracoronary calcium (98.8% and 99.4%, resp., *p*: 0.5). The results of other studies show the same trend [62, 63].

## 5. Coronary Computed Tomography Angiography (CCTA)

**Figure 2:**
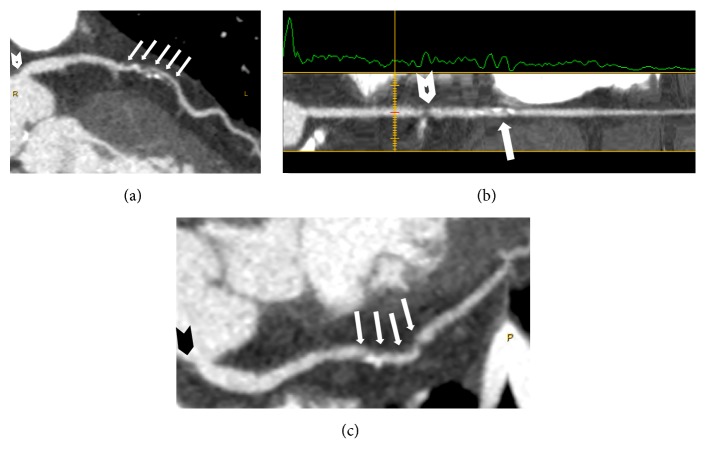
Computed tomography coronary angiography (CTCA) showing three-vessel disease. (a) Left anterior descending (LAD) artery. White arrows indicate a long, mixed, and severely obstructive plaque at proximal-middle LAD. White arrowhead shows a noncalcified, nonseverely obstructive plaque at the origin of left main artery (LM). (b) Right coronary artery (RCA). White arrow indicates a mixed and nonseverely obstructive plaque at middle RCA. White arrowhead shows a noncalcified, severely obstructive plaque at proximal RCA. (c) Left circumflex artery (LCx). White arrows indicate a long, mixed, and severely obstructive plaque at proximal-middle LCx. Black arrowhead shows a noncalcified, nonseverely obstructive plaque at the origin of LCx.

**Figure 3:**
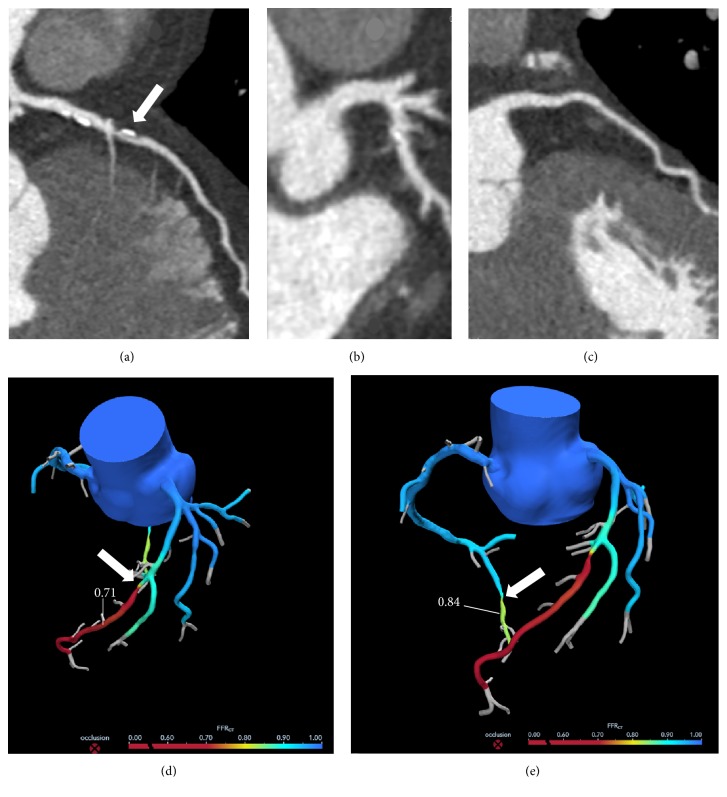
Computed tomography coronary angiography (CTCA) and fractional flow reserve CT (FFR_CT_). (a) Left anterior descending (LAD) artery. White arrow indicates a mixed, severely obstructive plaque at middle LAD. (b) Right coronary artery (RCA) with a small calcific spot. (c) Left circumflex (LCx) artery does not show atherosclerotic plaques. (d), (e) Graphic representation of FFR_CT_ calculation. (d) Following LAD stenosis (white arrow), the value is 0.71. (e) Following RCA stenosis (white arrow), the value is 0.84.

Recently, CCTA has emerged as a reliable noninvasive imaging tool for the identification of CAD [64–68]. Since is first steps, the technique has been characterized by a very high negative predictive value, whereas the positive predictive value has been growing progressively, mainly according to the improvement of many technical aspects [69]. The suboptimal positive predictive value and specificity of CCTA in assessing the coronary stenosis degree are mostly due to the “blooming” artifacts secondary to the presence of wall calcifications. In particular, the coronary arteries in diabetic subject are characteristically “small and calcific,” and this explains why the specificity of CCTA in this specific subset of patients may be particularly low (Figure 2). At the same time, the technological innovation has been taking a giant step towards the artifacts reduction by implementing different strategies throughout the process, from the premedication of the patient before scanning to the acquisition and analysis of the images [70–74]. Of note is that a new important and very attractive tool able to evaluate the functional value of a single stenosis, the fractional flow reserve CT (FFR_CT_), is not influenced by the presence of calcifications and thus is particularly reliable in diabetic population (Figure 3) [75–79]. Among all others, the employment of high definition techniques [80] allows high values of specificity and diagnostic accuracy (close to 90% and 95–98%, resp.).

Pivotal information is obtained by CCTA, specifically that obstructive and nonobstructive CAD are characterized by a higher prevalence in the diabetic population compared to normoglycemic patients and that a different plaque composition does exist [81–83].  Table 1 shows as a whole that among patients with DM, nonobstructive and obstructive CAD according to CCTA are associated with higher rates of all-cause mortality and major adverse cardiovascular events at follow-up, and this risk is significantly higher than that in nondiabetic subjects. Despite this, current European guidelines do not advise coronary CTA for risk assessment and suggest other noninvasive testing methods (nuclear imaging, echocardiography, and carotid ultrasound) in high risk diabetic patients [84]. Conversely, the latest American guidelines for detection and risk assessment of stable CAD state that calcium scoring and coronary CTA use “may be appropriate” in asymptomatic patients with high global risk [52, 85].

The fulcrum of noninvasive coronary assessment in diabetic population consists in its prognostic value. Numerous efforts have been made so far in order to add useful information on this debated topic.

Min et al. [86] evaluated the prognostic value of CCTA in a population of 400 asymptomatic diabetic patients without known history of CAD. This study showed that, after adjustment for CAD risk factors, the maximum stenosis, the number of coronary arteries involved, and the segment stenosis score are associated with increased risk of developing adverse cardiac events and had incremental power for predicting cardiac events over conventional risk factors. Moreover, the study revealed that CCTA confers incremental risk prediction, discrimination, and reclassification over CACS. Based on these results, CCTA seems to be very useful in risk stratification of asymptomatic diabetic patients at higher risk of developing adverse cardiac events. Halon et al. [87] examined the added value of CCTA over clinical risk scores of United Kingdom Prospective Diabetes Study (UKPDS) and coronary artery calcium in a population based cohort of 630 asymptomatic type 2 diabetics with no history of CAD assessed for coronary heart disease related events over 6.6 ± 0.6 years. Discrimination of all events was improved by addition of total plaque burden to the clinical risk and CACS combined and further improved by addition of an angiographic score.

Van Werkhoven et al. [44] confirmed the usefulness of CCTA in prognostic stratification of diabetic patients (*N* = 313) with known or suspected CAD compared to nondiabetic patients (*N* = 303). Authors found that DM (*p* < 0.001) and evidence of obstructive CAD (>50% coronary stenosis) (*p* < 0.001) were independent predictors of outcome. In particular and similarly to other bodies of evidence [88, 89], the presence of obstructive CAD is an important predictor of survival both in diabetic patients and in nondiabetic patients. Conversely, absence of atherosclerosis in CCTA is associated with excellent (100%) disease-free survival at a mean follow-up of 20 ± 5.4 months, confirming the known high predictive value of CT both in diabetic and in nondiabetic patients [90, 91].

Furthermore, the study conducted by Kim et al. [92] demonstrated that the duration of DM is significantly associated with the extent and the severity of CAD. Patients with a longer history of DM had higher levels of CACS, atheroma burden obstructive score, segment involvement score, and segment stenosis score (*p* < 0.001 for all). In addition, the severity of coronary stenosis clearly increases the incidence of adverse cardiac events, independently of other cardiovascular risk factors. On the basis of these considerations, authors suggest the introduction of CCTA screening in all patients with a history of DM > 10 years.

On the contrary, the study of Muhlestein et al. revealed that the use of CCTA as screening of asymptomatic diabetic patients did not reduce the incidence of mortality from all causes and nonfatal myocardial infarction. However, the value of this result could be resized taking into consideration the low incidence of adverse cardiac events in the study which reduces the statistical difference between the two groups [93]. Other than being unpowered, the study was biased by the fact that adequate care targets for risk factor reduction in most of patients assigned to receive aggressive therapy in CCTA group were not achieved. Moreover, the control group without CTA scanning also received good preventive medical treatment so that differences in therapy between the screened and nonscreened groups were subtle.

Recently, Kang et al. confirmed the prognostic value in long term of CCTA in a population of asymptomatic diabetics [94]. This study analyzed clinical outcome of 591 asymptomatic patients with type 2 DM undergoing CCTA showing that the survival free of cardiac events was 99.3 ± 0.7% in patients with normal coronary arteries, 96.7 ± 1.2% in those with nonobstructive CAD, and 86.2 ± 3.0% in those with obstructive CAD (log-rank *p* < 0.001). The present study confirmed that asymptomatic diabetic patients with normal coronary arteries or with nonobstructive CAD have an excellent clinical outcome even after five years, conversely to patients with obstructive CAD. An overview is given by a recent meta-analysis based on eight studies with a total of 6225 participants (56% male with average age of 61 years) and a mean follow-up of 20 to 66 months that evaluated the prognostic efficacy of CCTA in diabetic patients [95]. This meta-analysis concluded that CCTA is critical in identifying diabetic patients at high risk of CAD to be assigned to an aggressive modification of risk factors, glycemic control, and optimized medical therapy.

## 6. Therapeutic Perspectives

**Figure 4:**
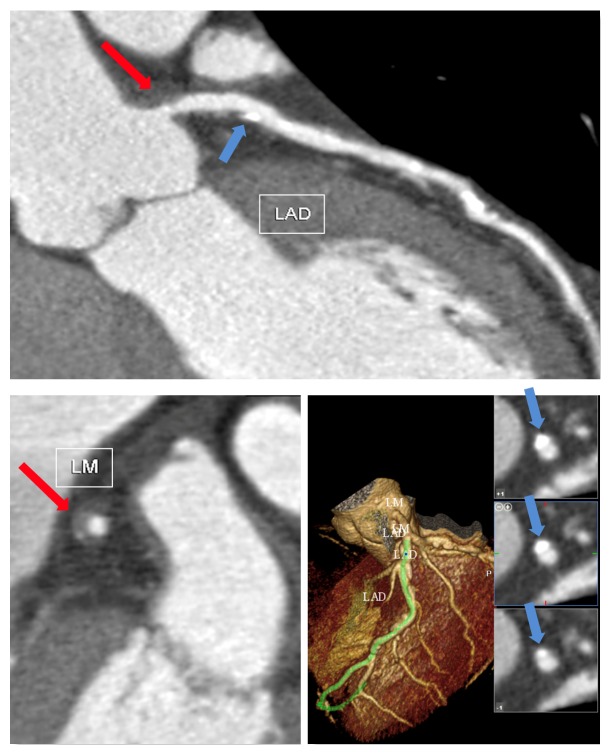
Diabetic patient with carotid disease, active inflammatory pattern, and multivessel coronary artery disease (CAD). The sole coronary artery calcium scoring (CACS) and coronary invasive angiography do not entirely represent the effective risk of the patients (CACS Agatston score 130 and stenosis of 30% of left main artery [LM]). The red arrow represents a very large, remodelled plaque with a little calcific component at the level of LM and thus a “high risk plaque.” The blue arrow represents a large, remodelled plaque with a bigger calcific component at the level of left artery descending (LAD). In this case the plaque does not generate any significant stenosis. Overall, these CAD features confer an incremental event risk.

At this point it is not incorrect to say that CCTA is able to distinguish between high and low risk diabetics patients, unveiling the presence of severe CAD. At the same time, CCTA can detail anatomic information of CAD features providing incremental power in the context of primary prevention of acute cardiac events [96]. The ability of this technique in revealing some vulnerability features of coronary plaque is known, including positive remodelling, presence of large plaque burden, and spotty calcification which increase the probability of plaque rupture and complication. Sometimes the “anatomic” high risk condition coexists in a “systemic” vulnerable context depicted by DM and kindled inflammatory state [97, 98] (Figure 4). A recent study [98] reported that diabetic subjects with increasing circulation levels of interleukins-6 and carotid artery disease had high probability of obstructive CAD and high risk plaques. Notwithstanding, the CV risk of the diabetic population is not uniform, and the vital and decisive pivot of the right identification of high risk subset of diabetics consists in impact on prophylactic therapy. The European Society of Cardiology guidelines recommend the consideration of aspirin use for primary prevention in patients at high risk with DM [17], while the Endocrine Society Clinical Practice guidelines recommend aspirin in patients with DM aged >40 years and whose 10-year CV disease risk is more than 10% [99]. Moreover, the existing risk charts tailored on patients with DM, such as the United Kingdom Prospective Diabetes Study and the Swedish National Diabetes Register [100, 101], need further validation for clinical applicability. Although noninvasive imaging tests demonstrated their value in risk stratification of diabetic subjects [102], no mention is made of the need for incorporating them in the diagnostic flow charts.

In a valuable attempt to correlate traditional CV risk factors with anatomic CAD features, Dimitriu-Leen et al. [103] prospectively studied a large asymptomatic diabetic population at high risk. On CCTA, 27% of these patients had no CAD. Considering patients with any CAD (73%), around half had obstructive CAD (more than 50% stenosis). Importantly, the study showed that the number and presence of risk factors were not associated with a higher frequency of CAD, except for hypertension. As a consequence, the authors underlined that CCTA could be pivotal in identifying which patients will benefit most from prophylactic prevention with aspirin. In this regard, it is necessary to keep in mind that aspirin is only useful if coronary atherosclerosis is present [104]. Notably, screening patients according to their CACS instead of exploring CAD on CCTA would result in undertreatment of 9% (diabetics with obstructive CAD) to 36% (diabetics with any CAD) of patients at high risk with DM who may benefit from therapy and this is in line with what other authors have shown [105]. The importance of well defining the high risk diabetic subjects worthy of prophylactic aspirin therapy derives from the evidence that the trials aimed at establishing its beneficial effect have been controversial and, particularly, 2 of those have failed in demonstrating significant reductions in CV events [106, 107]. Taylor et al. [108] in their analysis revealed the poor utility of statins use in diabetic population. This result, apparently paradoxical, highlights that diabetic patient should not necessarily be considered as a “coronaric” patient to be subjected to intensive medical therapy. Diabetic patients, in fact, in the presence of CACS < 10, have a brilliant prognosis, comparable with nondiabetic patients. Moreover, a substudy of the “coronary CT angiography evaluation for clinical outcomes: an international multicenter (CONFIRM) international registry” demonstrated in 4,706 patients with nonobstructive (less than 50% stenosis) CAD that prophylactic aspirin use was not associated with an improvement in all-cause mortality.

Although these bodies of evidence aim to demonstrate a rationale employment of prophylactic therapy, it seems clear that more comprehensive prospective studies, including inflammatory biomarker and polyvasculopathy assessment together with preventive treatment strategies, are warranted.

## 7. Conclusions

There is marked heterogeneity of risk among diabetic patients which has recently gained by scientific community. Clinical risk assessment, standard noninvasive imaging techniques, and CACS alone lack very accurate and tailored risk stratification at single level patient. Coronary computed tomography angiography represents a new technique able to detail CAD features providing diagnostic and prognostic information on asymptomatic type 2 diabetics. The direct consequences of this are that a significant proportion with no or very little coronary plaque are at negligible risk and others with more extensive plaque at considerably higher risk for an acute coronary event. Moreover, the prognostic prediction is refined with the consideration of plaque composition and with the assessment of inflammatory/polyvascular systemic involvement. In diabetics at low risk, the intensity of preventive medical therapy and frequency of follow-up may be reduced, particularly when there is intolerance to aspirin and high doses of statins or other prophylactic therapies. Afterwards, a stepwise approach of screening on the basis of cardiovascular risk factors and global clinical risk would allow characterization of a higher-risk group in which CACS followed by CTA is able to further risk stratification. In the setting of patients with more than 10 years of disease, a direct anatomical imaging strategy may allow the quick and reliable risk stratification of each patient. The identification of significant CAD in the context of a patient with DM could justify an intensive preventive regimen based on aspirin and, accordingly, on clinical conditions, statins, and antihypertensive drugs.

## Figures and Tables

**Table 1:** Randomized studies that investigated the prognostic power of CCTA in asymptomatic or stable patients with diabetic mellitus versus nondiabetics.

Authors/journal	Diabetics	Nondiabetics	Follow-up	Events in diabetics	Events in nondiabetics	Characteristic of CAD at CCTA	Univariate analysis in diabetics (HR)	Multivariate analysis in diabetics (HR)	Univariate analysis in nondiabetics (HR)	Multivariate analysis in nondiabetics (HR)
Van Werkhoven et al./Radiology 2010 [44]	*n* = 313	*n* = 303	20 ± 5.4 months	Total cardiac events (88)	Total cardiac events (45) *p* < 0.001	Obstructive	6.57 (*p* < 0.001)		16.29 (*p* < 0.001)	21.64 (*p* < 0.001)

Rana et al./Diabetes Care 2012 [6]	*n* = 3370	*n* = 6740	26 months	Death *n* = 108 (3.2%)	Death *n* = 115 (1.7%)	Nonobstructive		5.25		3.12 (*p* < 0.01)
						(1) Vessel disease		6.39		5.56 (*p* < 0.01)
						(2) Vessel disease		12.33		7.87 (*p* < 0.01)
						(3) Vessel disease		13.25		9.25 (*p* < 0.01)

Nadjiriet et al./Int J Cardiovasc Imag 2015 [45]	*n* = 108	*n* = 1379	66 ± 12.2 months	Cardiac events *N* = 10 (annual cardiac event rate 1.74%)		Number of lesions per patient (SIS)		3.0 (*p* = 0.047)		
						Segment stenosis score (SSS)		4.5 (*p* = 0.025)		
					Cardiac events *N* = 48 (annual cardiac event rate 0.64%)	Number of lesions per patient (SIS)				1.31 (*p* = 0.076)
						Segment stenosis score (SSS)				1.3 (*p* = 0.062)

Blanke et al./Jacc CI 2016 [46]	*n* = 1823	*n* = 1823	60 months	Death *n* = 246 (13.5%)	Death *n* = 136 (7.5%)		*RR for all-cause mortality in diabetics compared with propensity matched nondiabetics stratified according to extent/severity of CAD*			
						Nonobstructive	2.09 (*p* < 0.001)			
						Obstructive	1.95 (*p* < 0.001)			
						(1) Vessel disease	1.48 (*p* = 0.08)			
						(2) Vessel disease	2.45 (*p* = 0.003)			
						(3) Vessel disease	2.31 (*p* = 0.002)			

CAD: coronary artery disease; CCTA: coronary computed tomography angiography; HR: hazard ratio; RR: relative risk.
